# Extracorporeal Membrane Oxygenation After Norwood Surgery in Patients With Hypoplastic Left Heart Syndrome: A Retrospective Single-Center Cohort Study From Brazil

**DOI:** 10.3389/fped.2022.813528

**Published:** 2022-03-02

**Authors:** Rodrigo Freire Bezerra, Juliana Torres Pacheco, Victor Hugo Volpatto, Sônia Meiken Franchi, Rosangela Fitaroni, Denilson Vieira da Cruz, Rodrigo Moreira Castro, Luciana da Fonseca da Silva, José Pedro da Silva

**Affiliations:** ^1^Division of Congenital Heart Surgery, Hospital Beneficência Portuguesa de São Paulo, São Paulo, Brazil; ^2^Cardiac Intensive Care Unit, Hospital Beneficência Portuguesa de São Paulo, São Paulo, Brazil; ^3^Division of Cardiothoracic Surgery, University of Pittsburgh School of Medicine, Pittsburgh, PA, United States

**Keywords:** extracorporeal membrane oxygenation (ECMO), Norwood procedure, hypoplastic left heart syndrome (HLHS), cardiac arrest, survival analysis

## Abstract

**Background:**

Extracorporeal membrane oxygenation (ECMO) is increasingly being used to support patients after the repair of congenital heart disease.

**Objective:**

We report our experience with patients with a single functional ventricle who were supported by ECMO after the Norwood procedure, reviewing the outcomes and identifying risk factors for mortality in these patients.

**Methods:**

In this single-center retrospective cohort study, we enrolled 33 patients with hypoplastic left heart syndrome (HLHS) who received ECMO support after the Norwood procedure between January 2015 and December 2019. The independent variables evaluated in this study were demographic, anatomical, and those directly related to ECMO support (ECMO indication, local of initiation, time under support, and urinary output while on ECMO). The dependent variable was survival. A *p* < 0.05 was considered statistically significant.

**Results:**

The ECMO support was applied in 33 patients in a group of 120 patients submitted to Norwood procedure (28%). Aortic atresia was present in 72.7% of patients and mitral atresia in 51.5%. For 15% of patients, ECMO was initiated in the operating room; for all other patients, ECMO was initiated in the intensive care unit. The indications for ECMO in the cardiac intensive care unit were cardiac arrest in 22 (79%) of patients, low cardiac output state in 10 (18%), and arrhythmia in 1 patient (3%). The median time under support was 5 (2–25) days. The median follow-up time was 59 (4–150) days. Global survival to Norwood procedure was 90.9% during the 30-day follow-up, being 33.3% for those submitted to ECMO. Longer ECMO support (*p* = 0.004) was associated with a higher risk of death in the group submitted to ECMO.

**Conclusions:**

The mortality of patients with HLHS who received ECMO support after stage 1 palliation was high. Patients with low urine output were related to worse survival rates, and longer periods under ECMO support (more than 9 days of ECMO) were associated with 100% mortality. Earlier ECMO initiation before multiorgan damage may improve results.

## Introduction

Extracorporeal membrane oxygenation (ECMO) is being used to provide mechanical circulatory support for patients with congenital or acquired heart disease in cardiac failure when conventional medical management has failed. ECMO has been used with increasing frequency to support pediatric patients after repair or palliation of congenital heart disease (1–4). Indications for ECMO support in the postoperative period include refractory low cardiac output, persistent hypoxemia, arrhythmias, cardiac arrest, and failure to wean from cardiopulmonary bypass (1–3, 5).

The most common surgical strategy used as palliative therapy for hypoplastic left heart syndrome (HLHS) is the Norwood procedure, which has been used by our group for more than 35 years. The introduction of the right ventricle-to-pulmonary artery conduit strategy (6) had helped to improve our results even in an era when ECMO support was not available. Approximately 10–12% of the newborns undergoing the Norwood procedure require advanced circulatory support, and despite increasing the overall survival rate, ECMO use is associated with a high mortality rate in international publications (1–3, 5). Despite the increasing use of ECMO in such patients, only 31% survive until hospital discharge (2).

In Brazil, there is underreporting of the prevalence of congenital heart disease (6), and real experience in the surgical management of HLHS is lacking. Our group has a great history with the Norwood procedure, with patients treated since 1999 with good results (7, 8). To our knowledge, there are no published data regarding ECMO in HLHS in Brazil (9, 10).

The purpose of this study was to report our experience with functional single ventricle patients who were supported by ECMO after the palliative Norwood procedure. We reviewed the outcomes of ECMO support and identified risk factors for mortality in these patients.

## Materials and Methods

The current study followed the Strengthening the Reporting of Observational Studies in Epidemiology Statement (11). In this single-center retrospective cohort study, we included all patients with HLHS (International Classification of Diseases, 9th revision, code 746.7) who received ECMO support after a Norwood operation at *Hospital Beneficência Portuguesa de São Paulo* between January 2015 and December 2019. Exclusion criteria included HLHS patients who did not receive ECMO support and those who lacked information on records. All the clinical and surgical data for this cohort of patients were reviewed in the institutional database. The study was approved by the institution's Research Ethics Committee (2.925.146/2018).

The independent variables evaluated in this study were demographic variables (age, weight, sex, presence of aortic atresia, presence of mitral atresia, ascending aorta diameter, and atrial septal defect area) and variables related to ECMO support (location of ECMO initiation, indication of ECMO support, and duration of ECMO support). Location of ECMO included patients who received support in the cardiac intensive care unit (CICU) or in the operating room (OR). Indication of ECMO support included low cardiac output syndrome (LCOS), refractory arrhythmias, pulmonary hypertension, or cardiac arrest (extracorporeal cardiopulmonary resuscitation). Indications of extracorporeal cardiopulmonary resuscitation/cannulation included patients in CICU who were in cardiac arrest for 30 min maximum despite cardiopulmonary resuscitation maneuvers or those hemodynamically unstable after cardiac arrest. The clinical criteria for LCOS included the following: hypotensive shock despite vasoactive drugs—vasoactive inotropic score >35, metabolic acidosis (i.e., pH < 7.2 mmol/L), high lactate, central venous saturation <60%, oliguria (urine output <0.5 μg/kg/min), and end-organ hypoperfusion.

Additional analysis was performed focusing on neurological and renal outcomes. Neurological evaluation was done by transfontanelle ultrasonography and cranial computed tomography (CT), if necessary. Kidney function was evaluated according to the neonatal modified Kidney Diseases: Improving Global Outcomes (KDIGO) and urine output of the day on ECMO when a patient presented the peak creatinine level.

The dependent variable investigated in the present study was survival until the Glenn procedure. The demographic and ECMO-related data were compared between survivors and non-survivors of ECMO-supported patients.

Qualitative data were, depending on distribution, described as frequencies with percentages and quantitative data as mean or medians with ranges. All data were treated as non-parametric due to the size of the sample. We used Fisher's exact test to evaluate associations between qualitative data and the Mann–Whitney *U*-test to compare quantitative data among survivors and non-survivors. For survival, the time between the start of ECMO and death (event) or Glenn procedure (censorship) was considered—our policy is to keep all HLHS patients in the hospital until the second stage. We performed Kaplan–Meier survival analysis and used the log-rank test to determine significant differences in survival between the groups. A *p* < 0.05 was considered statistically significant. Data were analyzed and plotted using SPSS v25.

## Results

**Figure 1:**
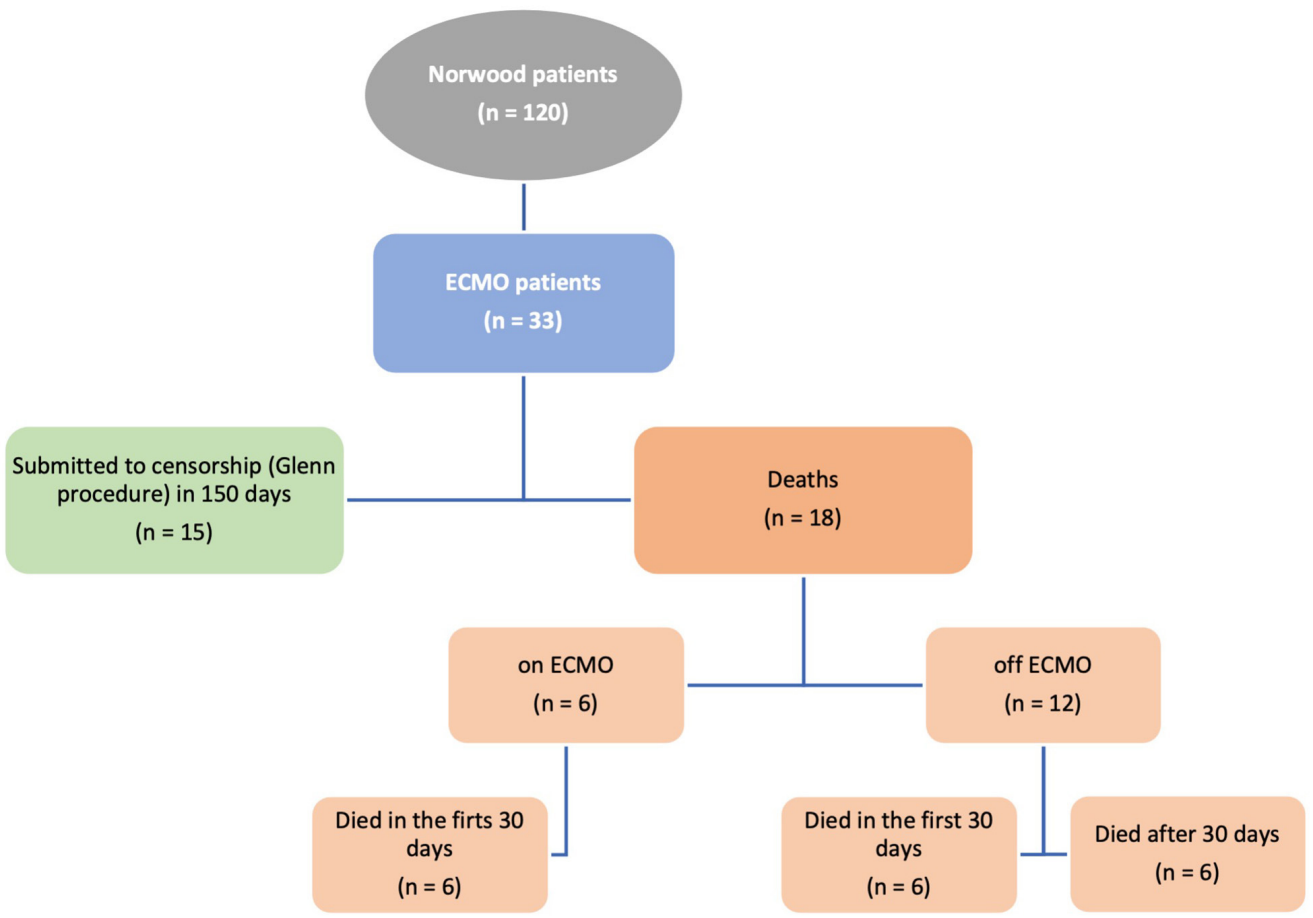
Flow chart of HLHS patients receiving ECMO assistance.

The total number of patients admitted for the Norwood procedure in this period was 120 patients. Of these patients, a total of 33 patients (28%) received ECMO support. The overall mortality of Norwood patients was 22% (18 patients on ECMO and 8 patients who did not receive extracorporeal support). Among the 18 deaths of patients who received ECMO, six (33.4%) had the support discontinued (died on ECMO). Of the 12 remaining patients (of ECMO patients), six were decannulated and died during the first 30 days of follow-up (Figure 1).

The median patient age of ECMO patients was 5 (2–70) days, median weight 3,115 (1,885–3,970) g, and 66.7% of patients were male. The median ascending aorta diameter was 2.5 (1.58–6.9) cm. ECMO was initiated in the OR for only 15% of patients due to low cardiac output (low urinary output, hypotension, and high lactate), low oxygen saturation, and/or high inotropic requirements; for all other patients (28 patients−85%), ECMO was initiated in the intensive care unit (ICU). The indications for ECMO in CICU were cardiac arrest in 22 (79%) of patients, low cardiac output state in 10 (18%), and arrhythmia in 1 patient (3%). The median time on ECMO support was 5 (2–25) days. The median follow-up was 59 (4–150) days.

We compared the demographic data between alive and dead patients (Table 1) to investigate possible risk factors among the basal patient characteristics. None of the independent variables related to patient demographics significantly differed between the two groups.

**Table 1:** Comparison of patient characteristics between alive and dead patients.

	**Alive (*n* = 11)**	**Dead (*n* = 22)**	***p*-value**
Age, days	5 (2–69)	3 (2–13)	0.1401^*^
Sex, male	8 (72.7%)	13 (59.1%)	0.7026^†^
Weight, g	3,115 (2,600–3,970)	3000 (2,500–3,800)	0.6178^*^
AAo diameter, cm	3.2 (1.6–6.6)	2.1 (1.7–6.9)	0.4398^*^
ASD area, mm^2^	13.3 (7.1–86.6)	20.0 (0.8–109.4)	0.6718^*^
ECMO initiated in OR	2 (14.3%)	3 (15.8%)	0.99^†^
ECMO initiated in CICU	12 (85.7%)	16 (84.2%)	0.99^†^
ECMO duration	5 (2–8)	7.5 (4–25)	0.004^*^
Urine output	1.5 (0.55–5.4)	0.21 (0.04–2.3)	0.001^*^

*Aao, ascending aorta; ASD, atrial septal defect*.

*Data are expressed as median (range) or n (%)*.

* *Mann–Whitney U-test*;

† *Fisher's exact test*.

**Figure 2:**
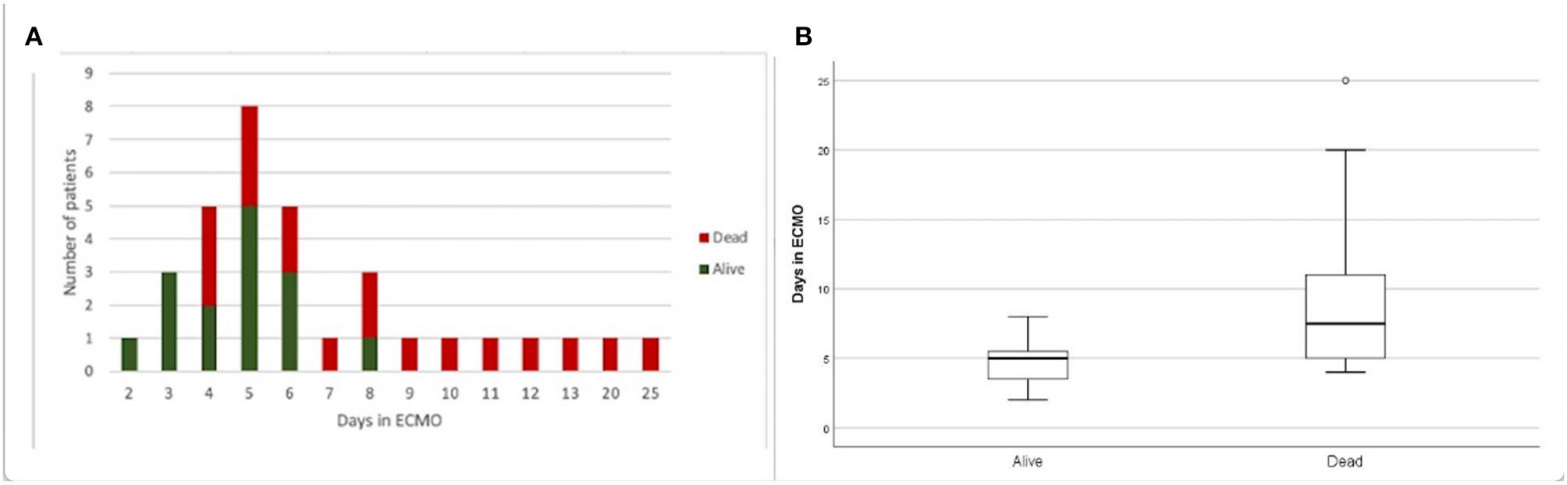
Survival according to time under ECMO support. **(A)** Frequency of dead and alive patients submitted to different durations (2–25 days) of ECMO support. **(B)** Comparison of days under ECMO support for dead and alive patients. Tukey boxplot, whiskers represent 1.5 interquartile range. *Mann–Whitney *U-*test, *p* = 0.004.

We analyzed survival rates according to the time under ECMO support (Figure 2). All patients (100%) survived when ECMO support lasted 2 and 3 days, 40% with 4 days, 63.5% with 5 days, 60% with 6 days, 0% with 7 days, and 33.3% with 8 days. All seven patients who received ECMO support for 9 days or longer died (Figure 2A). Also, only 1 of 11 patients survived beyond 6 days of ECMO. The comparison of survivors and non-survivors showed a significant difference between the groups (Figure 2B). The ECMO support duration (median) was 5 days [95% confidence interval (CI): 3.8–5.5] for the group of patients that was alive and 7.5 days (95% CI: 6.2–11.8) for the group who had died (Mann–Whitney *U*-test, *p* = 0.004).

**Figure 3:**
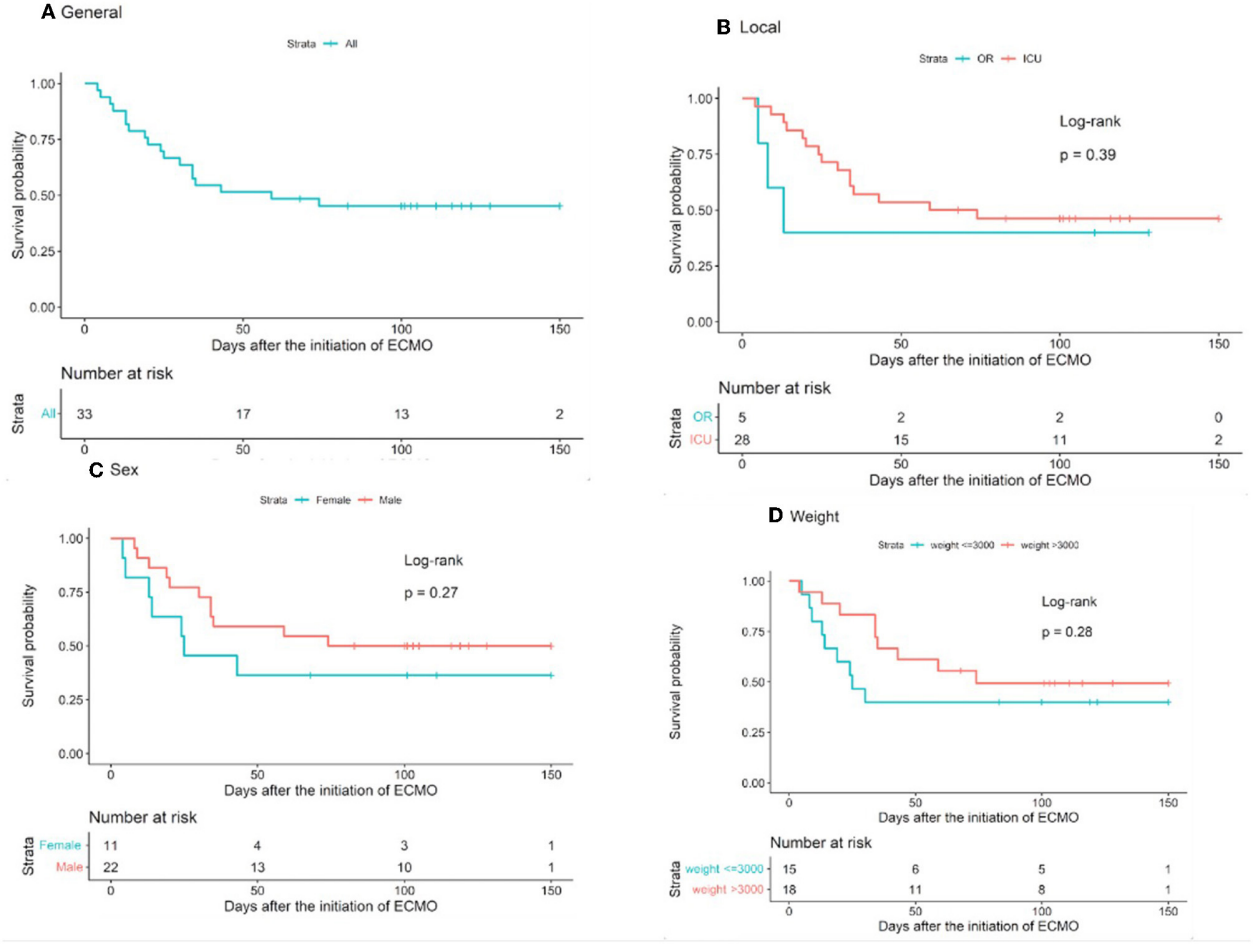
Survival curves. **(A)** General and according to **(B)** location of ECMO initiation, **(C)** patient sex, and **(D)** patient weight. Error bars indicate standard error of mean.

Of the 33 ECMO patients, 15 (45%) were alive during the follow-up. We also performed a survival analysis, which showed 33.3% overall survival until censorship (Glenn procedure). Survival analysis was expressed using the Kaplan–Meier survival curves and was used to evaluate different variables as risk factors for death, including ECMO indication, location of ECMO initiation, sex, and weight (Figure 3). Patients with cardiac arrest as an indication for ECMO had lower median survival, 43 days when compared with those which the indication was not cardiac arrest, 74 days; however, on the complete follow-up period, there were no differences in regard to survival rate related to the indication (*p* = 098). The median survival for patients with ECMO initiated in the OR was 13 and 59 days for those to whom ECMO was initiated in the ICU. Male patients also demonstrated higher median survival than female patients (74 vs. 25 days); however, this difference was not significant when considering the complete follow-up period (*p* = 0.271). Lastly, important differences in the median survival rates were also noted based on patient weight, with 25 days for those weighing <3 kg and 74 days for those weighing >3 kg. However, considering the complete follow-up period, weight was not a risk factor for death (*p* = 0.280). In summary, most of these variables seem to play an important role early during follow-up, but in the long term, they were not demonstrated to be associated with survival.

Additional analysis was performed focusing on the presence of valvar atresia. We evaluated the presence of isolated and combination aortic and mitral atresia as a risk factor for death. We did not find that any of the variables were associated with the survival rate.

**Figure 4:**
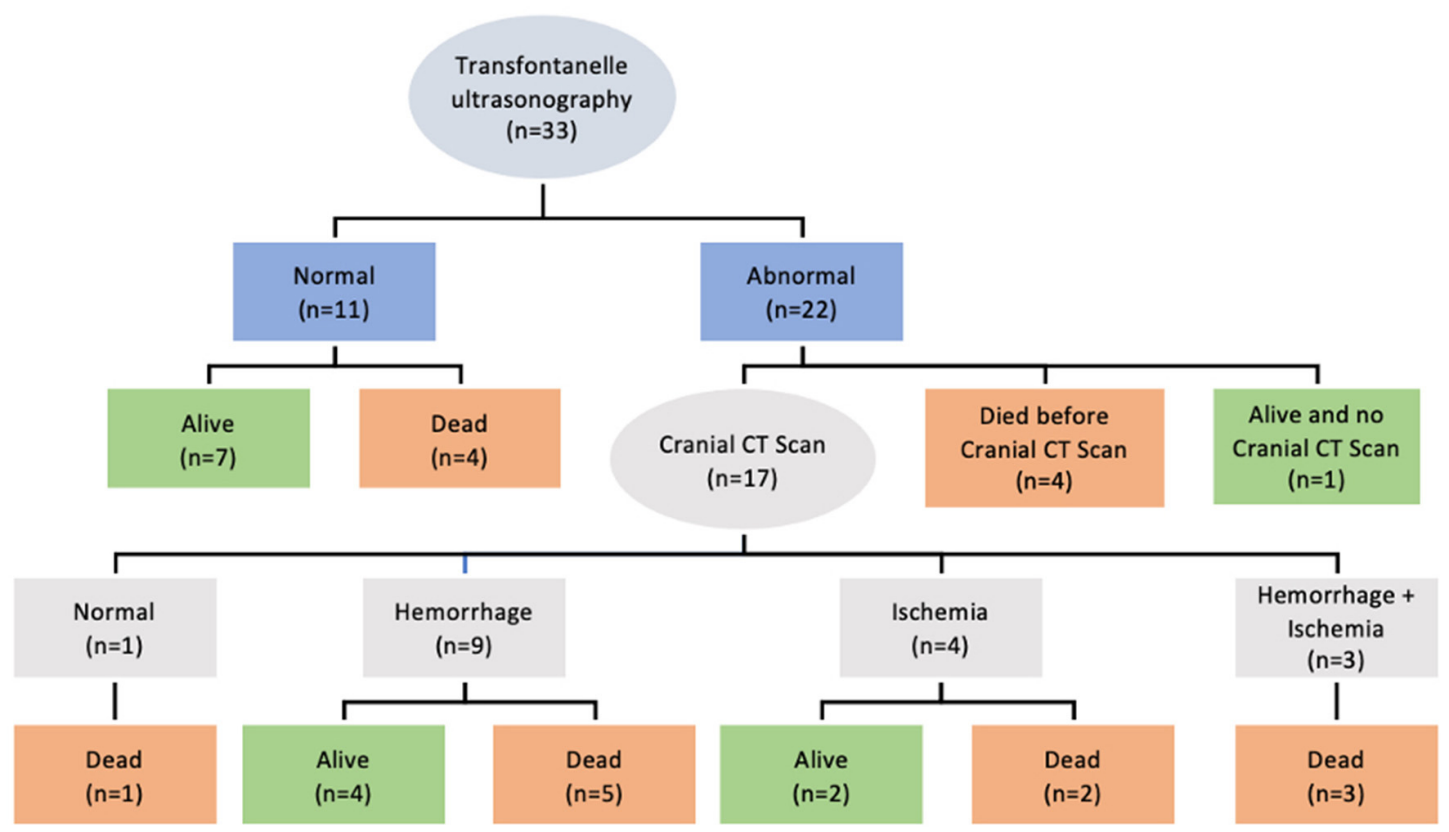
Neurological findings in patients receiving ECMO assistance.

All patients submitted to ECMO were screened for neurological outcomes by transfontanelle ultrasonography. A third of the patients were considered normal, and the other two-thirds were suspected of presenting some degree of neurological findings. Four of these patients died before evaluation with cranial CT, one patient was still alive but had no CT scan performed and only one was considered free from neurological abnormalities. All the other patients presented with hemorrhage, ischemia, or both. Figure 4 details the findings and outcomes of the neurological evaluation. The risk of death was higher in patients with neurological findings during screening with transfontanelle ultrasonography than in patients considered to be free from neurological abnormalities but with no statistical significance (*p* = 0.407).

**Figure 5:**
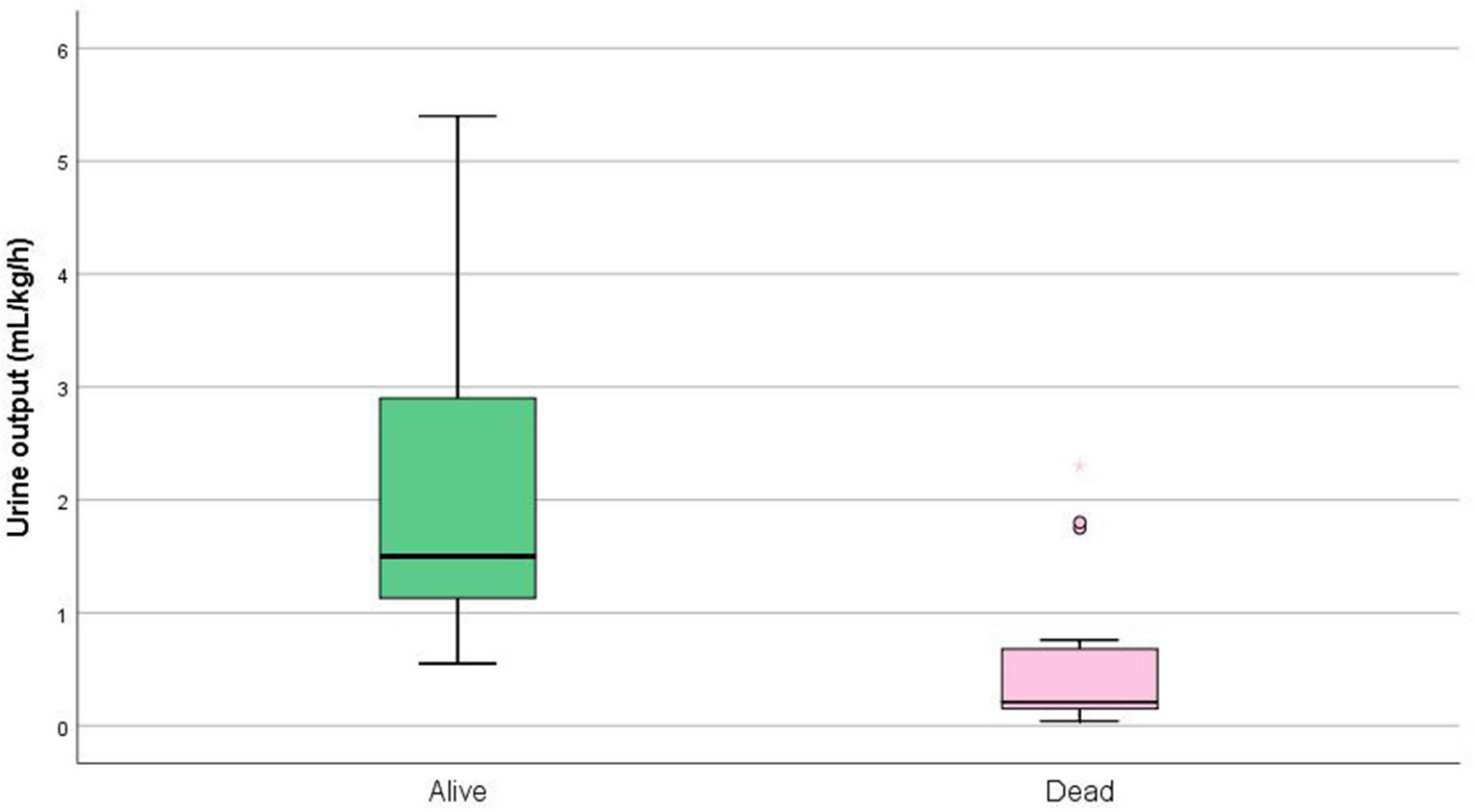
Kidney function according to urine output in patients receiving ECMO. Tukey boxplot, whiskers represent 1.5 interquartile range (*p* = 0.001).

According to the neonatal modified KDIGO, 100% of our patients went from KDIGO stage 0 (before ECMO) to KDIGO stage 3 (while on ECMO). An analysis of kidney function using urine output (Figure 5 and Table 1) found a worse outcome for those patients who presented a lower urine output.

## Discussion

The right ventricle in HLHS may present a lower performance reserve after Norwood surgery. Therefore, early postoperative ECMO support can theoretically benefit the global result after Norwood palliation. It is a fundamental tool to both promote adequate multiorgan perfusion and allow myocardial recovery. We found that Norwood patients who received advanced circulatory support had high mortality. We have not found a relationship between the mortality rate and the location of ECMO initiation or ECMO indication. However, longer periods under ECMO support were related to worse survival rates. The presence of renal impairment may be related to a delayed indication and initiation of ECMO, with the low cardiac output period causing end-organ damage (12).

Although no universal consensus exists as to which definition of acute renal failure to use, neonatal modified KDIGO is currently the most used criteria (13, 14). However, all patients were receiving renal replacement therapy (KDIGO stage 3), whereas on ECMO (according to our ECMO routine), what compromised our renal function analysis. The only evaluation that remained, although poor, as it suffered too many interferences (volemic status, use of diuretics, and presence of dialysis), was patient's urine output, which showed that patients who died presented a lower urine output.

Our 4-year experience demonstrated 33.3% global overall survival in Norwood patients receiving ECMO, which is an excellent result and is in concordance with previous reports of short- and mid-term outcomes for patients requiring ECMO support after Norwood surgery (15–17). We attribute these numbers to our ECMO team and experience with Norwood patients. According to Mesher et al. (18) neonates with single-ventricle heart disease represent one of the most challenging groups of patients to manage. Other Brazilian publications on HLHS are represented by a few cases and are more related to the hybrid approach (19, 20).

The Society of Thoracic Surgeons (1, 21, 22) reports a higher number of Norwood cases needing ECMO, and the reported survival to hospital discharge is variable (28–64%) (2). A recent analysis of data from the Pediatric Heart Network Single-Ventricle Reconstruction emphasized that patients who ultimately require ECMO after a Norwood procedure are the same patients who face an increased risk of mortality after the surgery (5). However, variables that are usually implicated in worse overall survival, such as birth weight, age, ascending aorta diameter, and mitral/aortic atresia, did not differ between survivors and non-survivors in our experience.

As HLHS patients submitted to the Norwood procedure represent the largest group of neonates who require ECMO for cardiac support (23), we investigated the risk factors related to these poor outcomes. Rather than identifying characteristics of HLHS patients that require ECMO, we analyzed risk factors for poor outcomes once on ECMO support.

Regarding the moment when ECMO was initiated, most Norwood patients started ECMO support in the ICU during the early postoperative period before the sternum is closed, as already reported in the literature (2). A few patients (15%) started ECMO in the OR due to the impossibility of weaning from cardiopulmonary bypass. For almost all patients, ECMO was initiated due to cardiac arrest or low cardiac output state while in the ICU. A potential association between the location of ECMO initiation and survival was evaluated, but the statistical analysis did not find one. Arrhythmia was responsible for ECMO initiation in just one patient. In the analysis of Hoskote et al. (5), severe refractory tachydysrhythmia, either as a consequence of or the cause of poor ventricular function, was a significant predictor of poor survival because the combination of LCOS and negative inotropic drugs used to control the tachydysrhythmia are poorly tolerated after the Norwood stage 1 procedure.

We found that longer periods under ECMO support were a risk factor for poor prognosis. Sherwin et al. (2) and Khorsandi et al. (24) also demonstrated that few Norwood patients survive more than 9 and 10 days on ECMO support, respectively, and our mortality rate after 9 days was 100%. To a certain extent, ECMO could be understood as a marker of illness severity rather than a true cause of death.

In addition to the duration of support, ECMO-related complications, such as neurologic injury and renal failure, are also considered risk factors for death (2). In our series, this was also demonstrated. The high morbidity caused by multiorgan failure and sepsis after decannulation is a significant problem, resulting in long intensive care and hospital stays. Multiple organ system failure, defined as the failure of at least two organ systems, was an independent risk factor for death in such patients. Montgomery et al. (25), in a review of 59 children who received ECMO after cardiopulmonary bypass over a 9-year period, reported that progressive multiple organ system dysfunction and development of nosocomial infection were significantly associated with death. Two-thirds of the patients in our series received ECMO after cardiac arrest, which can increase the risk of multiorgan failure.

We recognize that our study has some limitations. The relatively small cohort limited our ability to detect differences between groups and to perform any subgroup analyses. Together with the unavailability of data regarding additional variables, such as the details of anatomic variants of HLHS and the shunt type used in stage 1 palliation, precluded the possibility of performing multivariate analysis and identifying confounding factors. Thus, important factors influencing mortality may be missing. Furthermore, other important outcomes, such as long-term survival, neurological and functional outcomes, and quality of life, were not investigated in the present study. Renal function analysis was also compromised by our renal replacement therapy routine while on ECMO, as earlier mentioned. However, the data reported by the Extracorporeal Life Support Organization Registry are not specific for the analysis of ECMO outcomes for patients with HLHS after stage 1 palliation, which justifies the reporting of our series despite the limitations mentioned earlier.

In this study, ECMO support was lifesaving for a group of patients that otherwise would have died. Therefore, ECMO remains an important resource, although highly technical and expensive, when facing adverse hemodynamics in Norwood patients in the immediate postoperative period. After analyzing our data, we have tried to avoid ECMO initiation after cardiac arrest, being more premature on ECMO indication. We now have a perfusionist and a cardiovascular surgeon (ECMO team) 24 h on call to facilitate ECMO initiation without delay and have also become more aggressive on indicating ECMO discontinuation after a week of support.

Strategies are needed to improve survival until hospital discharge to justify the continued use of ECMO in this patient group, especially as critical care unit and hospital stays are prolonged. Further research exploring other strategies, such as lowering the clinical threshold for ECMO indication, early transition to the pathway of prolonged mechanical circulatory assistance with newer devices, and heart transplantation, should investigate the potential to improve outcomes in this high-risk population.

## Conclusions

The mortality of patients with HLHS who received ECMO support after stage 1 palliation was high. Longer periods under ECMO support (more than 9 days of ECMO) were associated with 100% mortality. Earlier ECMO initiation before multiorgan damage may improve results.

## Data Availability Statement

The raw data supporting the conclusions of this article will be made available by the authors, without undue reservation.

## Author Contributions

All authors listed have made a substantial, direct, and intellectual contribution to the work and approved it for publication.

## Conflict of Interest

The authors declare that the research was conducted in the absence of any commercial or financial relationships that could be construed as a potential conflict of interest.

## Publisher's Note

All claims expressed in this article are solely those of the authors and do not necessarily represent those of their affiliated organizations, or those of the publisher, the editors and the reviewers. Any product that may be evaluated in this article, or claim that may be made by its manufacturer, is not guaranteed or endorsed by the publisher.
